# Development of a multivariable prognostic prediction model for skin tears in older nursing home residents

**DOI:** 10.1038/s41598-025-95944-5

**Published:** 2025-04-03

**Authors:** Monira El Genedy-Kalyoncu, Bettina Völzer, Jan Kottner

**Affiliations:** Charité – Universitätsmedizin Berlin, corporate member of Freie Universität Berlin and Humboldt-Universität zu Berlin, Institute of Clinical Nursing Science, Charitéplatz 1, 10117 Berlin, Germany

**Keywords:** Skin tears, Prediction models, Risk factors, Long-term care, Prevention, Geriatrics, Risk factors

## Abstract

Skin tears are traumatic wounds and are among the most prevalent skin conditions in older adults, particularly those in long-term care facilities. These injuries can lead to complications such as infection, pain, reduced quality of life, and increased healthcare costs. This study aimed to identify risk factors for skin tear development in nursing home residents aged 65 years or older and to develop a predictive prognostic model. A secondary data analysis was performed on long-term care nursing home residents ≥ 65 years who participated in a cluster-randomized controlled clinical trial conducted in Berlin, Germany, from April 2019 to June 2021. A total of 101 residents were included. At week 12, 19 residents (18.8%) developed at least one skin tear. The best-fit predictive model identified lower Body Mass Index, lower Barthel Index scores, presence of xerosis cutis on the legs, and regular corticosteroid use as significant risk factors for skin tear development. The model demonstrated good discriminatory ability (area under the curve: 0.823), with sensitivity and specificity rates of 73.7% and 74.4%, respectively. These risk factors could help identify at-risk individuals, enabling targeted preventive measures. However, the model requires validation in a prospective cohort to confirm its applicability in clinical practice.

**Supplementary Information:**

The online version contains supplementary material available at 10.1038/s41598-025-95944-5.

## Introduction

According to the International Skin Tear Advisory Panel (ISTAP), a skin tear is defined as “a traumatic wound caused by mechanical forces, including removal of adhesives. Severity may vary by depth (not extending through the subcutaneous layer)”^1^. Skin tears can occur on any area of the body but are predominantly located on the upper and lower extremities^2^. In vulnerable populations, such as critically ill or hospitalized individuals, patients with chronic diseases, and long-term care nursing home residents, skin tears are one of the most prevalent skin conditions, particularly with increasing age^1,3^.

In aged long term-care settings, the reported prevalence of skin tears varies significantly, ranging from 3% in Belgium to between 6.3% and 11% in Germany, and 14.7–20.8% in Canada^3–7^. Skin tears are a significant concern across health settings due to their high risk of serious medical complications, including infection, pain, and reduced quality of life. These injuries also lead to high treatment costs, labor-intensive wound care, and increased hospitalizations, placing a considerable burden on healthcare providers and systems^2^.

Worldwide, the aging population is expected to grow substantially, with projections indicating that the proportion of individuals being 65 years or older will increase from less than 10% in 2021 to approximately 17% by 2050^8^. In Europe, approximately 30% of this population relies on long-term care services^9^. The aging process is associated with various physiological changes, including the development of chronic diseases, dependency on care, and functional decline, all of which also affect the skin. Environmental factors, such as UV exposure and smoking, along with lifestyle factors like repeated harmful skincare practices, further compromise skin integrity^10^.

Given this context, an increase in age-associated conditions, including skin tears, is anticipated, and the implementation of effective prevention and management strategies is essential^11^. In institutional long-term care settings, skin care is an integral part of nursing practice, and nursing interventions can significantly influence skin health. Nurses, particularly those working with older individuals, must be aware of the risk of skin tear development, and their potential consequences. Awareness and early identification of individuals at risk are crucial for the timely initiation of appropriate preventive nursing measures^12^.

The existing literature on the identification of risk factors for skin tears in aged care and corresponding prediction models and their predictive abilities has been compared and summarized by Rayner et al.^13^ and Fan et al.^14^. Characteristics such as prior history of skin tears, senile purpura, advanced age, and presence of hematoma have been shown to increase skin tear risk, but evidence regarding long-term care settings is sparse, and follow-up periods were often very short.

The objective of this study was to identify risk factors associated with the development of skin tears in aged long-term care nursing home residents and to develop a prognostic model to identify individuals who are most at risk.

## Methods

### Source of data

A secondary data analysis was conducted. We utilized data from an investigator-initiated, investigator-blinded, exploratory cluster-randomized controlled trial conducted in 17 institutional aged long-term care facilities with 314 participating residents in the federal state of Berlin, Germany, from April 2019 to June 2021^15^. The primary aim of the underlying clinical trial was to assess the effects of implementing an evidence-based skincare program to prevent common adverse skin conditions, including skin tears, pressure ulcers, incontinence-associated dermatitis, xerosis cutis, and intertrigo in residential long-term care settings. The clinical trial was approved by the ethics committee at the Charité—Universitätsmedizin Berlin, Germany, (approval number: EA1/243/18) and was prospectively registered at ClinicalTrials.gov (Identifier: NCT03824886). The study was developed and conducted in accordance with the principles stated in the Declaration of Helsinki and its subsequent amendments, as well as the Guidelines for Good Clinical Practice (ICH Topic E6 (R1)). Informed consent was obtained from all participants and/or their legal guardians prior to study participation. The study was funded by public funds from the Federal Ministry of Education and Research (BMBF, grant number 01GL1801). The study protocol and main results of the clinical trial have been published elsewhere^15,16^.

### Participants

#### Eligibility criteria at participant level

(I) Permanent residency in the nursing home at the time of baseline data collection; (II) age of 65 years or older with no upper age limit; (III) being substantially care-dependent according to the German Social Code Book XI (care level II or higher); (IV) written informed consent provided by the resident or legal representative. Due to ethical considerations, residents at the end of life were excluded. Additionally, residents requiring special dermatological treatments at baseline were excluded to avoid possible contamination between medical treatments and skincare interventions.

#### Eligibility criteria at cluster level

(I) Nursing homes located in the federal state of Berlin, Germany; (II) capacity of at least 70 beds; and (III) the presence of a pressure ulcer prevention standard in the institution. Each nursing home represented one cluster.

#### Eligibility criteria for the dataset

The complete original dataset of the underlying clinical trial was available without restrictions. For this analysis, all clusters and participants from the (I) control group were included, provided that participants had both a (II) baseline visit and (III) a week 12 follow-up visit, and (IV) did not have a skin tear at the baseline examination.

Only data from the control group were utilized to prevent potential contamination from the intervention, ensuring that the identified risk factors for skin tears were not influenced by the skincare program implemented in the intervention group^17^.

### Outcome

The outcome predicted by the model was the development of new skin tears 12 weeks after baseline. Skin tears were assessed through comprehensive head-to-toe skin examinations conducted by dermatologists at baseline and at the 12-week follow-up visit. All assessments were performed by study physicians who were members of the research team and blinded to group allocation. As the prediction model was not planned at the time of the study’s conduct, physicians were also blinded regarding the outcome to be predicted. Physicians were specifically trained for the trial and had experience examining older residents in nursing home settings. This standardized assessment approach was chosen to ensure consistent and reliable data collection. The assessment of skin tears was based on the classification criteria established by the ISTAP^18,19^.

### Predictors

#### Data collection procedures

Comprehensive demographic and health-related data, including physical examinations, were collected for all participants from primary and secondary sources prior to cluster randomization to prevent any influence of the intervention. Demographic and health information was extracted from medical records and/or obtained through interviews with the participants. For participants with cognitive limitations, missing information was obtained by interviewing relatives, legal representatives, or nursing staff. If data remained missing after this process, it was recorded as missing in the database. The data collection process was standardized and uniformly conducted across all clusters. Standardized case report forms were used, and data were subsequently entered into an electronic case report form. All data were collected exclusively by researchers, study nurses, and clinicians who were members of the study team.

#### Variables and operationalization

Demographic variables (collected at baseline): The variable age was expressed in years at the time of the baseline visit. The variable sex was categorized as male or female. Duration of residency was expressed in months and corresponds to the time period between admission to the nursing home and the baseline visit. Body Mass Index (BMI) was calculated using the formula: weight [kg]/ height [m^2^]. BMI was analyzed as a continuous variable and categorized into two categorical variables: Overweight (BMI ≥ 25) and underweight (BMI < 18.5). These cut-offs were based on the definition of the World Health Organization (WHO)^20^.

Cognitive and Functional Assessments (collected at baseline): The Global Deterioration Scale (GDS), ranging from 1 (no cognitive impairment) to 7 (very severe cognitive impairment), was applied to measure the severity of cognitive impairment^21^. Functional assessments were conducted using the Barthel Index , ranging from 0 to 100, with higher scores indicating greater functional independence, to measure physical function related to daily activities^22^. Pressure ulcer risk was assessed with the Braden Scale, ranging from 6 to 23 points, where 18 or higher indicates low risk, 15–17 moderate risk, 13–14 high risk, and 12 or lower very high risk^23^. Care level was classified according to the German Social Code Book XI.

Medical Conditions (collected at baseline): Diseases were coded according to the International Classification of Diseases 11th Revision (ICD-11)^24^. The variable dementia included all subjects with diagnoses starting with the first three ICD-11 characters ‘6D8’, encompassing various forms of dementia, including Alzheimer’s disease. The variable Paralytic symptoms included all diagnoses starting with the first three ICD-11 characters ‘MB5’, covering various paralytic syndromes such as Tetra-/Para-/Hemiplegia. The variable hemiparesis included residents with the diagnosis coded as 6B60.6.

Medication (collected at baseline): Regular medication was coded using the Anatomical Therapeutic Chemical (ATC) classification system and categorized according to the therapeutic subgroups, such as medications affecting the renin-angiotensin system (ATC code C09) and corticosteroids (ATC code H02)^25^. The variable Polypharmacy was defined as the daily intake of five or more different pharmaceutical agents^26^.

Skin Conditions (collected at baseline and week 12): Dry skin (xerosis cutis; ICD-11 ED54) was categorized according to the Overall Dry Skin Score (ODS)^27^. Incontinence-associated dermatitis (ICD-11 EK02.22) was categorized in accordance with the Ghent Global Incontinence-Associated Dermatitis Categorisation Tool in urinary and/or faecal incontinent subjects^28,29^. Pressure ulcers (ICD-11 EH90) were categorized according to the National Pressure Ulcer Advisory Panel/European Pressure Ulcer Advisory Panel/Pan Pacific Pressure Injury Alliance (NPUAP/EPUAP) classification^30,31^. Pressure ulcers were included only from NPUAP/EPUAP category II and above. Intertrigo (ICD-11 EK02.2) was diagnosed according to ICD-11 as an irritant contact dermatitis of the skin folds caused by repetitive shearing forces of skin on skin due to friction, sweating or contact with body fluids^24^. Skin tears were classified according to ISTAP (Type I–III)^18^.

For all skin conditions, the presence or absence of the condition was considered as a binary variable (yes/no); location and severity were not included. Due to the nearly universal prevalence of xerosis cutis in the study population, this condition was included in the analysis only if present on the arms or legs. To capture this accurately, two separate binary variables were created to indicate the presence of xerosis cutis specifically on the arms and the legs.

### Sample size

No formal sample size calculation was performed for this prediction model. Instead, the full dataset from the original cluster randomized controlled trial was utilized, including all participants and clusters that met the inclusion criteria. This approach was chosen to maximize the use of all available information from the participants and nursing homes involved in the study, thereby maximizing the number of events (skin tears) and enhancing the reliability of the prediction model.

### Statistical analysis methods

Continuous variables were described using means and standard deviations (SD) or medians and interquartile range (IQR: 25th and 75th percentiles). Categorical variables and the outcome of newly developed skin tears at 12 weeks after baseline were presented as numbers (n) and proportions (%). The intracluster correlation coefficient (ICC) for skin tears was calculated using the one-way analysis of variance (ANOVA) method to assess the degree of clustering within nursing homes. Missing data were labeled in the tables and excluded from the analyses. All calculations were conducted using IBM SPSS Version 28.

#### Predictor selection

Demographic, health, and functional characteristics of residents who developed a new skin tear at week 12, and those without skin tears, were compared using mean differences for continuous variables or odds ratios (ORs) for categorical variables, along with corresponding 95% confidence intervals (CIs). Group differences with a *p*-value of < 0.05 (two-sided), ORs ≤ 0.5 or ≥ 2, or notable mean differences, were considered as possible predictors. Univariate binary logistic regression analyses were conducted to identify factors associated with skin tear development. Variables with a *p*-value of < 0.05 in the univariate analysis were considered statistically significant and included as candidate predictors. Additionally, clinically relevant variables that could be assessed in clinical practice were considered. Variables identified in the literature as potential predictors for skin tears were also included, regardless of their statistical significance.

To limit collinearity and ensure a parsimonious model with the smallest number of relevant independent variables, Pearson’s correlation coefficients (r) were calculated among all predictor variables, and a correlation matrix was constructed. Variables with correlation coefficients of |r|> 0.8 were considered highly correlated. In such cases, only one variable from each highly correlated pair was retained for further modeling steps.

#### Model derivation

All candidate predictors were included in a multivariable logistic regression. A stepwise backward elimination process using the Wald statistic was employed, where predictors not meeting the significance threshold (*p* < 0.05) were sequentially removed. The model was refined by reintroducing clinically relevant variables that had been excluded, and assessing their impact on the model’s performance. The final model was selected based on a combination of statistical significance, clinical relevance, and model fit indicators.

Multicollinearity was further assessed by calculating the Variance Inflation Factor (VIF) for each predictor variable; predictors with VIF values greater than 10 were excluded. At each iteration of the model, VIF values were monitored to ensure that multicollinearity was not present.

#### Internal validation and performance assessment

To account for potential overfitting and to obtain unbiased estimates of predictive performance, internal validation was performed using bootstrapping. A total of 1000 bootstrap samples were generated by resampling the original dataset with replacement. For each bootstrap sample, the entire model development process was repeated, including predictor selection and model fitting.

The model’s discriminatory ability was assessed using the Area Under the Curve (AUC) of the Receiver Operating Characteristic (ROC) curve. AUC values range from 0 to 1, with 0.5 indicating no discrimination and 1.0 indicating perfect discrimination; higher values indicate better model performance. In addition to AUC, sensitivity (proportion of true positives correctly identified) and specificity (proportion of true negatives correctly identified) were computed to assess the model’s predictive accuracy. A threshold of 0.7 was established as an acceptable performance level for both sensitivity and specificity metrics.

To evaluate the model’s calibration, calibration plots were generated to compare the predicted probabilities of skin tears with the observed outcomes. This ensured that the predicted risks aligned with the actual occurrence of skin tears, providing confidence that the model’s risk estimates accurately reflect the true probabilities. Calibration was further assessed by comparing the predicted and observed outcomes, with a *p*-value of > 0.05 indicating good agreement.

#### Generalized estimating equations

To account for clustering at the nursing home level, a generalized estimating equations (GEE) model was used to develop the final set of predictors for the model. This approach accommodates the nested data structure inherent in nursing homes, where residents within the same facility may share similar environmental or care-related factors. The GEE model employed an independent correlation structure, specifying the nursing home identifier as the clustering variable. A binomial distribution with a logit link function was used. Variables were entered into the GEE model, and a stepwise backward and forward elimination process was performed.

### Assessment of risk of bias and applicability

The risk of bias and applicability were assessed using the Prediction Model Risk of Bias Assessment Tool (PROBAST)^32^. While PROBAST is typically used in systematic reviews of prediction models, its application here aligns with the recommendations from the TRIPOD-Cluster statement’s explanation and elaboration document^33^. Structured risk of bias assessments are suggested when repurposing existing datasets that were not originally intended for prediction model development. Given that the dataset in this study was not initially collected for the purpose of model development but repurposed for this analysis, PROBAST was used to evaluate potential bias.

The PROBAST risk of bias assessment consists of 4 domains (participants, predictors, outcome, and analysis) and the applicability assessment includes 3 domains (participants, predictors, and outcome), with a total of 20 signaling questions. Each question can be answered with “yes,” “probably yes,” “no,” “probably no,” or “no information.” A domain where all signaling questions are answered as “yes” or “probably yes” is judged as having low risk of bias or concerns regarding applicability. An answer of “no” or “probably no” on one or more questions per domain flags the potential for bias, while “no information” indicates insufficient data to make a judgment. The overall risk of bias for each domain is determined as 'low,' 'high,' or ‘unclear’ based on the combined evaluation of all signaling questions. If all domains were rated as low risk, the overall risk of bias was judged to be low; however, if any domain was rated as high risk, the overall risk was classified as high.

## Results

### Participants and clusters

**Fig. 1:**
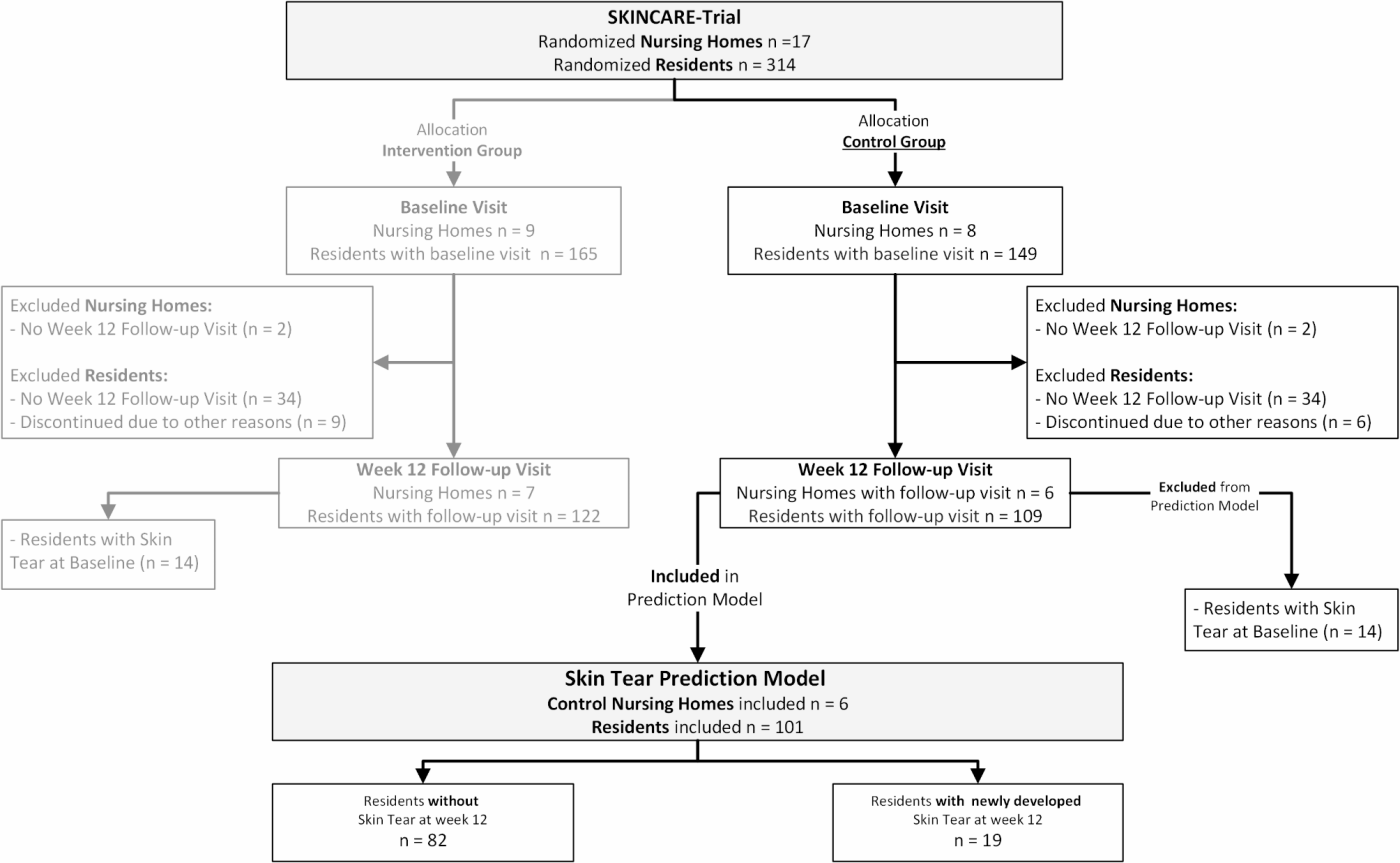
Flow chart data selection.

At baseline, 149 participants allocated to the control group were enrolled across 8 clusters. However, due to the COVID-19 pandemic, 2 clusters comprising 34 residents were not available for the 12-week follow-up. Additionally, 6 participants from the 6 remaining clusters could not be followed up for other reasons (e.g., death or being at the end of life). Consequently, follow-up data at week 12 were available for 109 residents across 6 clusters. Of these 109 residents, 8 participants presented with a skin tear at baseline, and were therefore excluded from this analysis. This left a final sample of 101 participants from 6 clusters for analysis. No additional clusters or participants were excluded due to missing data or other reasons beyond the established eligibility criteria. Figure 1 provides a detailed flow chart of the data selection process, illustrating which data were included in this analysis from the complete dataset of the underlying clinical trial.

### Sample characteristics

Table 1 provides an overview of the baseline characteristics of the analyzed sample, categorized by the presence or absence of a newly developed skin tear at week 12. The mean age of participants was 84.7 years (SD 7.2), and the majority were female (66.3%). The mean BMI was 26.9 kg/m^2^ (SD 5.4). At the week 12 follow-up visit, 19 out of 101 residents developed a skin tear, resulting in a cumulative incidence of 18.8%. The ICC for skin tears at week 12 was 0.172.

**Table 1:** Baseline Characteristics. Baseline characteristics of control group residents for variables included in the development of the prediction model.

Continuous variables	Newly developed skin tear at week 12 n = 19	No skin tear at week 12 n = 82	Mean difference (95% CI)	*p*-value
Age [years], mean (SD)	84.2 (7.8)	84.8 (7.1)	0.61 (− 3.21 to 4.04)	0.745
BMI [kg/m^2^], mean (SD)	22.5 (3.9)	27.3 (5.7)	**4.84 (2.60–7.23)**	** < 0.001**
Duration of residency [months], mean (SD)	17.7 (16.9)	30.3 (29.4)	**12.52 (2.55–21.71)**	**0.015**
Braden Scale [score], mean (SD)				
Total Score	14.8 (3.0)	16.5 (3.4)	**1.99 (0.59–3.47)**	**0.008**
Mobility Subscore	2.3 (0.7)	2.6 (0.8)	0.27 (− 0.07 to 0.62)	0.170
Barthel Index [score], mean (SD)				
Total Score	33.4 (23.9)	43.9 (25.1)	**10.48 (**− **1.60 to 21.39)**	0.078
Standing/ Mobility Subscore	6.1 (4.9)	7.4 (4.8)	1.39 (− 1.00 to 3.80)	0.244
Transfer Subscore	8.4 (5.5)	9.2 (4.9)	0.73 (− 1.99 to 3.17)	0.572

Categorical variables			Odds ratio (95% CI)	
Sex, n (%)				
Female	10 (52.6)	57 (69.5)	**0.49 (0.16–1.48)**	0.161
Male	9 (47.4)	25 (30.5)		
Underweight (BMI < 18.5), n (%)	3 (15.8)	2 (2.4)	**7.50 (1.33–34.79)**	**0.016**
Overweight (BMI ≥ 25.0), n (%)	6 (31.6)	54 (65.9)	**0.24 (0.07–0.71)**	**0.006**
Care level, n (%)				
II and III	12 (63.2)	58 (70.7)	0.71 (0.56–2.40)	0.519
IV and V	7 (36.8)	24 (29.3)	1.41 (0.46–4.00)	0.519
Smoker/former smoker, n (%)	10 (66.7)*	38 (50.0)*	**2.00 (0.63–8.53)**	0.237
Incontinence type, n (%)				
Faecal incontinence	11 (57.9)	31 (37,8)	2.26 (0.75–7.44)	0.109
Urinary incontinence	13 (68.4)	70 (85.4)	1.37 (0.59–3.29)	0.435
Dementia, n (%)	10 (52.6)	27 (32.9)	**2.26 (0.79**–**7.23)**	0.108
Medication, n (%)				
Medication influencing the renin-angiotensin system	8 (42.1)	49 (59.8)	**0.49 (0.17**–**1.37)**	0.162
Corticosteroids	1 (5.3)	5 (6.1)	1.30 (0.61–8.68)	0.811
Polypharmacy (≥ 5)	17 (89.5)	74 (90.2)	0.92 (0.19–3.12)	0.919
GDS Stages, n (%)				
GDS 1–3	10 (52.6)	64 (78.0)	1.31 (0.25–4.28)	**0.024**
GDS 4–7	9 (47.4)	18 (22.0)	**3.20 (1.00**–**10.30)**	**0.024**
Skin conditions, n (%)				
Xerosis cutis arms	17 (89.5)	64 (78.0)	**2.39 (0.63**–**7.29)**	0.260
Xerosis cutis legs	18 (94.7)	71 (86.6)	**2.79 (0.53**–**5.32)**	0.322
Incontinence-associated dermatitis	3 (15.8)	19 (23.2)	0.62 (0.15–2.14)	0.482
Pressure ulcer	1 (5.3)	3 (3.7)	1.46 (0.61–10.93)	0.747
Intertrigo	6 (31.6)	26 (31.7)	1.34 (0.28–7.23)	0.991
Other medical conditions, n (%)				
Diabetes	6 (31.6)	40 (48.8)	**0.49 (0.12**–**1.32)**	0.175
Hemiparesis	3 (15.8)	5 (6.1)	**2.89 (0.63**–**17.29)**	0.159
Paralytic symptoms	1 (5.3)	2 (2.4)	**2.22 (0.83**–**13.30)**	0.514

*CI* confidence intervals, *BMI* body mass index, *GDS* global deterioration scale.

*Smoker status was available for 15/19 individuals with skin tears and 76/82 individuals without skin tears.

Bold values indicate statistical significance (*p* < 0.05), odds ratios of ≤ 0.5 or ≥ 2, or relevant mean differences considered in the model development process.

### Risk of bias assessment and applicability

All PROBAST domains were judged to be at low risk of bias. The underlying RCT included a representative sample of nursing home residents aged 65 and older. The inclusion/exclusion criteria were appropriately defined. All predictors were uniformly defined and assessed across participants, and the predictor assessments were conducted without knowledge of the outcome data. Additionally, all predictors included in the model are readily available in clinical practice. The outcome (skin tears) was classified using the standardized ISTAP definition. None of the predictors used in the model were involved in determining the outcome. Assessors were blinded to predictor information, as the decision for prediction modeling was made after data collection. The 12-week follow-up period provided an appropriate time interval between predictor assessment and outcome determination. The PROBAST assessment, including detailed judgement of each domain, is available in Appendix 1.

### Model development

#### Predictors

A total of 30 variables were included in the initial analysis (Table 1). In the group comparison, 6 baseline characteristics emerged as potential candidate predictors for the development of skin tears by week 12, based on significance. Participants who developed skin tears had a significantly lower BMI than those who did not (mean difference 4.84, 95% CI 2.60–7.23, *p* < 0.001). More specifically, being underweight (BMI < 18.5) was significantly associated with skin tears (OR 7.50, 95% CI 1.33–34.79, *p* = 0.016), while participants who were overweight (BMI ≥ 25) were less frequently found in the group that developed skin tears (OR 0.24, 95% CI 0.07–0.71, *p* = 0.006). Residents who developed skin tears had a shorter duration of residency in the nursing home (mean difference 12.52, 95% CI 2.55–21.71, *p* = 0.015), and those with lower Braden Scale scores were more likely to be at risk (mean difference 1.99, 95% CI 0.59–3.47, *p* = 0.008). Higher GDS scores (between 4 and 7) were more common in residents who developed skin tears (OR 3.20, 95% CI 1.00–10.30, *p* = 0.024).

The Barthel Index Total Score showed a substantial mean difference between groups (mean difference 10.48, 95% CI − 1.60 to 21.39, *p* = 0.078), and was therefore included in further analysis, as lower scores were associated with skin tears.

Additional variables were included based on their ORs (≤ 0.5 or ≥ 2). Higher frequencies of skin tears were associated with dementia (OR 2.26, 95% CI 0.79–7.23, *p* = 0.108), xerosis cutis on the arms (odds ratio 2.39, 95% CI 0.63–7.29, *p* = 0.260) and legs (OR 2.79, 95% CI 0.53–5.32, *p* = 0.322), hemiparesis (odds ratio 2.89, 95% CI 0.63–17.29, *p* = 0.159), paralytic symptoms (OR 2.22, 95% CI 0.83–13.30, *p* = 0.514), and smoking status (OR 2.00, 95% CI 0.63–8.53, *p* = 0.237). Conversely, lower frequencies of skin tears were observed among participants who were taking medications influencing the renin-angiotensin system (OR 0.49, 95% CI 0.17–1.37, *p* = 0.162), those with diabetes (OR 0.49, 95% CI 0.12–1.32, *p* = 0.175), and female participants (OR 0.49, 95% CI 0.16–1.48, *p* = 0.161).

#### Univariate logistic regression

Results of the univariate logistic regression analysis for each potential predictor are shown in Table 2, reflecting their individual effects on the outcome. Lower Braden Scale total scores at baseline were associated with increased odds of developing skin tears (OR 0.842, 95% CI 0.724–0.980, *p* = 0.026). Similarly, a decrease of one unit in BMI was linked to a 22% increase in the odds of skin tear development (OR = 0.780, 95% CI 0.675–0.901, *p* < 0.001). When BMI was categorized, underweight individuals had a significantly higher risk, with a 7.5-fold increase in the odds of developing skin tears (OR = 7.50, 95% CI 1.16–48.56, *p* = 0.035). Conversely, individuals categorized as overweight (BMI ≥ 25) had a 76% reduction in the odds (OR = 0.24, 95% CI 0.08–0.70, *p* = 0.009). Residents classified in GDS stages 4–7 were at a 3.2-fold higher risk of developing skin tears (OR 3.20, 95% CI 1.13–.07, *p* = 0.029).

**Table 2:** Univariate candidate predictor analysis.

Continuous variables		Odds Ratio (95% CI)	*p*-value
Age [years]		0.988 (0.922–1.059)	0.738
BMI [kg/m^2^]		0.780 (0.675–0.901)	** < 0.001**
Duration of residency [months]		0.978 (0.954–1.003)	0.080
Braden scale [score]			
Total score		0.842 (0.724–0.980)	**0.026**
Mobility subscore		0.627 (0.3211.223)	0.171
Barthel index [score]			
Total score		0.983 (0.962–1.004)	0.104
Standing/mobility subscore		0.941 (0.846–1.046)	0.259
Transfer subscore		0.971 (0.879–1.073)	0.568

Categorical variables	Reference category		
Female sex	Male sex	**0.49 (0.18–1.35)**	0.165
Underweight (BMI < 18.5)	Absence	**7.50 (1.16–48.56)**	**0.035**
Overweight (BMI ≥ 25.0)	Absence	**0.24 (0.08–0.70)**	**0.009**
Care level IV or V	Care level II/III	1.41 (0.50–4.01)	0.520
Smoker/former Smoker	Non-smoker	2.00 (0.63–6.41)*	0.243*
Incontinence type			
Urinary incontinence	Absence	0.741 (0.18–3.00)	0.674
Faecal incontinence	Absence	**2.26 (0.82–6.24)**	0.115
Dementia	Absence	**2.26 (0.82–6.22)**	0.113
Medication			
Medication influencing the renin-angiotensin system	Absence	**0.49 (0.18–1.35)**	0.167
Corticosteroids	Absence	1.46 (0.14–14.89)	0.748
Polypharmacy ($\ge$ 5)	Absence	0.92 (0.18–4.72)	0.919
GDS 4–7	GDS 1–3	**3.20 (1.13–9.07)**	**0.029**
Skin conditions			
Xerosis cutis arms	Absence	**2.39 (0.51–11.33)**	0.272
Xerosis cutis legs	Absence	**2.80 (0.34–23.04)**	0.341
Incontinence-associated dermatitis	Absence	0.62 (0.16–2.36)	0.486
Pressure ulcer	Absence	1.46 (0.14–14.89)	0.748
Intertrigo	Absence	0.994 (0.34–2.91)	0.991
Other			
Diabetes	Absence	**0.49 (0.17–1.40)**	0.180
Hemiparesis	Absence	**2.89 (0.63–13.33)**	0.174
Paralytic symptoms	Absence	**2.22 (0.19–25.86)**	0.524

Univariate binary logistic regression for possible associations between variables with the development of skin tears.

*CI* confidence intervals, *BMI* body mass index, *GDS* global deterioration scale.

*Smoker status was available for 15/19 individuals with skin tears and 76/82 individuals without skin tears.

Bold values indicate statistical significance (*p* < 0.05) or odds ratios of ≤ 0.5 or ≥ 2.

For duration of residency, each additional month was associated with a 2% reduction in the odds of developing a skin tear (OR 0.978, 95% CI 0.954–1.003, *p* = 0.080). Individuals with fecal incontinence demonstrated over twice the odds of developing skin tears (OR 2.26, 95% CI 0.82–6.24, *p* = 0.115), and those with dementia had a 2.26-fold increased risk (OR 2.26, 95% CI 0.82–6.22, *p* = 0.113).

The Braden Mobility Score indicated a potential increase in risk, with reduced mobility linked to a 37% increase in the odds of skin tear development per unit decrease (OR = 0.627, 95% CI 0.321–1.223, *p* = 0.171). Smoking status also trended toward increased risk, with smokers showing a twofold increase in the odds of developing skin tears (OR = 2.00, 95% CI 0.63–6.41, *p* = 0.243), though this result was not significant.

Other variables, such as the use of medication influencing the renin-angiotensin system (OR 0.49, 95% CI 0.18–1.35, *p* = 0.167), xerosis cutis on the arms (OR 2.39, 95% CI 0.51–11.33, *p* = 0.272), and xerosis cutis on the legs (OR 2.80, 95% CI 0.34–23.04, *p* = 0.341), showed some association with skin tear risk, though none of these findings reached statistical significance. Detailed results are presented in Table 2.

To assess relationships between predictors and address potential collinearity, a correlation analysis was conducted. The correlation matrix is shown in Appendix 2. The Braden Scale total score was strongly correlated with its Mobility sub-score (r = 0.912), the Barthel Index total score (r = 0.840), and its sub-scores for Standing/Mobility (r = 0.848) and Transfer (r = 0.790). The Barthel Index also correlated with the Braden Mobility sub-score (r = 0.811). Fecal incontinence showed a positive correlation with GDS stages 4–7 (r = 0.671). To avoid collinearity, variables measuring the same construct, such as BMI and its categories (e.g., Underweight or Overweight), were not included together in the models. Instead, only one variable per construct was used to prevent redundancy and improve model stability.

#### Multivariable logistic regression

Initially, all selected predictors were included in the multivariable logistic regression model. Among highly correlated pairs, only one variable from each pair was retained. The Barthel Index was preferred over the Braden Scale and its sub-scores due to its stronger association with the outcome. Although the Barthel Index did not reach statistical significance (*p* = 0.114), it remained in the final model of the Backward Wald.

GDS 4 to 7 was retained in place of fecal incontinence, which was removed early in the backward selection process. Similarly, xerosis of the legs was kept instead of xerosis of the arms, as it showed greater consistency throughout the selection process. BMI was used as a continuous variable, as it consistently showed significance across all models, rather than its categorical forms (underweight/overweight). It consistently emerged as a significant predictor across all models (Backward Wald: *p* = 0.001; Forward Selection: *p* = 0.002; Bidirectional Stepwise: *p* = 0.002).

#### GEE analysis

To account for clustering at the nursing home level, candidate predictors were analyzed using GEE. The initial set of variables selected for GEE analysis, after considering collinearity, redundancy and relevance, included: age, BMI, duration of residency, Barthel Index at baseline, sex, smoker status, dementia, medication (angiotensin), corticosteroid use, GDS (stages 4–7), diabetes, hemiparesis, paralytic symptoms, IAD, pressure ulcers, intertrigo, and xerosis of the legs at baseline.

Multicollinearity was assessed using the Variance Inflation Factor (VIF). All variables demonstrated acceptable levels of multicollinearity, with VIF values below the common threshold of 5. BMI (VIF = 1.697) and GDS (stage 4–7) (VIF = 2.138) showed the highest values but remained within acceptable limits.

Through a stepwise backward elimination and forward selection process, predictors that did not meet the significance threshold (*p* > 0.05) were excluded. The final model included four significant predictors of skin tear development at week 12: BMI, use of corticosteroid medications, presence of xerosis on the legs, and Barthel Index score (Table 3).

**Table 3:** Variables included in the final model adjusted for clusters.

Variable [category]	Regression Coefficient (β)	Standard Error	Wald χ^2^	df	*p*-value	95% CI for β
BMI	− 0.286	0.054	27.901	1	< .001	− 0.393 to -0.180
Barthel index total score	− 0.027	0.012	4.995	1	0.025	− 0.051 to -0.003
Corticosteroid medication use [present]	0.981	0.333	8.709	1	0.003	0.330–1.633
Xerosis on legs [present]	1.795	0.748	5.763	1	0.016	0.330–3.261

β: regression coefficient; Wald χ^2^: Wald chi-square statistic; df: degree of freedom; CI: Confidence interval; BMI: Body Mass Index.

#### Model performance

**Fig. 2:**
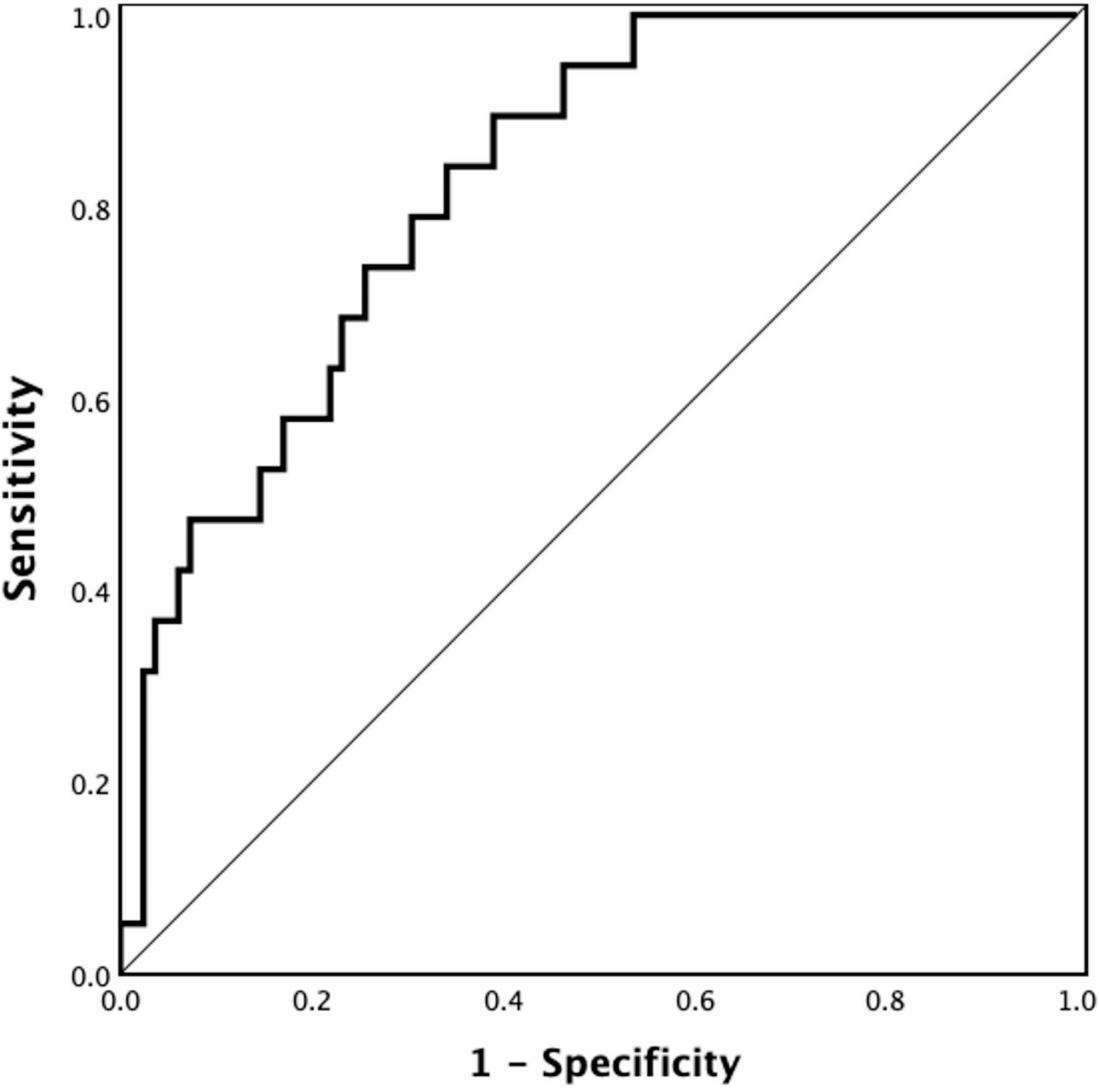
Receiver Operating Characteristic (ROC) curve of the final prediction model.

The ROC analysis of the final predictive model yielded an AUC of 0.823 (95% CI 0.703–0.943), indicating strong discrimination between residents who developed skin tears and those who did not (Fig. 2).

Sensitivity and specificity were calculated at different cutoff probabilities. Initially, a threshold probability of 0.7 was applied, resulting in a specificity of 98.8% but a sensitivity of only 5.3%. To address the imbalance between sensitivity and specificity at this initial cutoff, an exploration of alternative cutoff values (0.1, 0.3, 0.5) was conducted. Furthermore, a balanced cutoff value (≈ 0.175) was identified, achieving approximately equal sensitivity and specificity. This balanced cutoff yielded a sensitivity of 73.7% and a specificity of 74.4%. Table 4 summarizes the performance metrics across these cutoff values.

**Table 4:** Results of receiver operating characteristic (ROC) analysis for different classification cutoffs.

Classification cutoff probability	Sensitivity (%)	Specificity (%)
0.1	94.7	46.3
0.3	52.6	82.9
0.5	47.4	86.0
0.7	5.3	98.8
≈ 0.175*	73.7	74.4

*Optimal Balanced Cutoff: Represents the cutoff probability where sensitivity and specificity are most balanced.

**Fig. 3:**
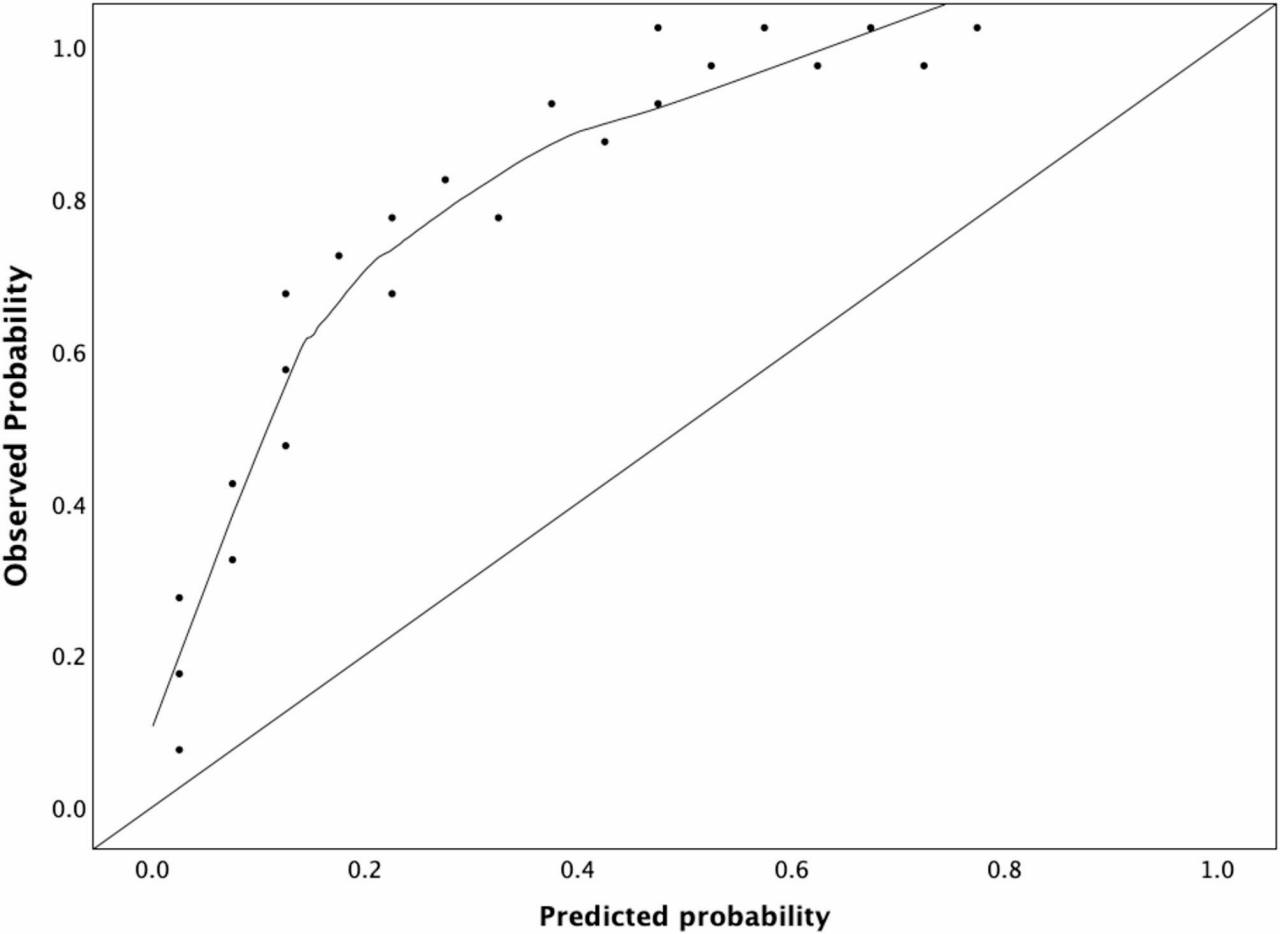
Calibration plot for the final prediction model.

The calibration plot demonstrated good alignment between predicted probabilities and observed outcomes in the lower-risk groups (e.g., predicted probability of 0.09 vs. observed proportion of 4.00%) (Fig. 3). However, the model showed a tendency to overestimate the risk of skin tears in higher probability groups (e.g. predicted probability of 0.57 vs. observed proportion of 10.00%). The Hosmer–Lemeshow goodness-of-fit test was non-significant (χ^2^ = 3.80, df = 8, *p* = 0.875), indicating good overall calibration of the model. These findings suggest that, while the model provides accurate risk estimates for individuals with lower predicted probabilities, there is a tendency to overpredict risk in higher-risk categories.

## Discussion

This study aimed to identify risk factors for the development of skin tears in aged long-term care residents by conducting a secondary analysis of control group data from a cluster-randomized controlled trial. The final predictive model included BMI, corticosteroid use, xerosis on the legs at baseline, and Barthel Index scores as significant predictors of skin tear development 12-weeks after baseline.

### Predictors in the final model

A lower BMI was associated with an elevated risk of skin tear development. Underweight individuals have reduced subcutaneous fat and skin elasticity, making them more susceptible to effects of mechanical forces^34^ that may cause skin tears. The observed protective effect in individuals with a higher BMI (76% reduction in odds) further highlights the importance of maintaining a sufficient, but not excessive, body mass to prevent such injuries. The use of corticosteroid medications was another significant predictor, increasing the likelihood of skin tear development. The regular use of corticosteroids can impair skin integrity, making it more susceptible to trauma, and has been linked to skin tear development^35^. The presence of xerosis on the legs was also identified as a significant risk factor. Xerosis, particularly common in populations 65 years or older, is closely associated with impaired skin barrier function^36^. An association between dry skin and skin tear development has been shown before^37^ and it is considered a modifiable risk factor for skin tear development^35^. The Barthel Index, a measure of functional independence, was a significant predictor in this study. Residents with lower scores, indicating higher care dependency, had a greater risk of skin tears. This association may reflect increased handling, friction, and trauma during assistance with activities such as dressing or mobility^38^.

### Model performance

The final predictive model demonstrated good discriminatory ability, with an AUC of 0.823, indicating strong performance in distinguishing between residents who developed skin tears and those who did not. The model calibration was further assessed using a calibration plot and the Hosmer–Lemeshow test, which was non-significant, indicating satisfactory calibration overall. However, some overestimation of risk was observed in higher-risk categories, suggesting the need for further refinement to improve prediction accuracy in these groups.

The sensitivity (73.7%) and specificity (74.4%) at a balanced cutoff probability of approximately 0.175 indicate that the model effectively identifies true positives while minimizing over-prediction in lower-risk residents. However, sensitivity decreased at higher cutoff values, which could limit the model’s utility in settings that prioritize high sensitivity where avoiding false negatives is critical.

The events per variable ratio was approximately 4.75, which is below the recommended threshold of 10–15 events per variable, increasing the potential risk for overfitting. However, bootstrapping techniques were used to account for this limitation, helping to improve model robustness.

### Differences from previous prediction models

Many of the earlier skin tear prediction models focused on factors such as prior skin tears, hematoma, or senile purpura^14^—variables that were not systematically collected in the underlying clinical trial of this analysis. In this analysis, the BMI consistently emerged as a significant predictor across both univariate and multivariable models, demonstrating an independent effect on skin tear risk throughout. The other variables only became significant within the multivariable framework in this study, suggesting their effects are more context-dependent and reliant on interactions with other variables.

When reviewing the existing literature on skin tear prediction models in older adults, BMI has typically not been included as a predictor^14^. Some prediction model studies did not collect or report BMI data, making direct comparisons difficult. Furthermore, their eligibility criteria may have excluded individuals with extremely high or low BMI, thereby narrowing the overall BMI range. In contrast, the sample used for this analysis had minimal eligibility restrictions, which may have resulted in a broader and more representative study population, thereby potentially making the BMI effect more pronounced. Moreover, previous models often assessed dryness in a generalized manner. In this analysis, however, dryness was specifically examined on the legs or arms, potentially amplifying its role as a distinct predictor of skin tears.

### Implications for clinical practice

The identification of modifiable risk factors, such as xerosis and BMI, has practical implications for care in long-term care settings. Implementing routine skincare protocols, including the regular use of emollients for residents with dry skin, could improve skin barrier function and reduce the incidence of skin tears. Evidence suggests that the application of topical leave-on products helps to reduce skin tear incidence^15,39^ and may also strengthen the dermo-epidermal junction^40,41^. Additionally, monitoring residents with low BMI could address their increased vulnerability to skin tears, and improvement of the nutritional status might help to prevent them^42^. The inclusion of the Barthel Index as a predictor seems to support the finding that skin tear development is associated with general care dependency and functional limitations^43^. Strategies to minimize friction and shear during bed transfers or repositioning, especially for residents with lower Barthel Index scores, seem to be helpful.

### Limitations

This study has several limitations. It is important to note that the original trial was not specifically designed to develop a prediction model for skin tears. As a secondary analysis of an existing clinical trial dataset, the study was not statistically powered for this purpose. Consequently, the statistical power and precision of our estimates, particularly for detecting interactions between variables, may have been limited by the relatively small sample size and the number of clusters. The ICC of 0.172 indicates, that the effects of the individual nursing homes were also high.

Several relevant risk factors commonly included in other skin tear prediction models—such as senile purpura, history of previous skin tears and falls^14^—were not collected in the underlying trial. This limits direct comparisons with existing models and may have affected the comprehensiveness of the predictive model. Furthermore, because the study focused exclusively on residents from a specific geographic region and care setting, the generalizability of the findings to broader populations or other settings is limited.

## Conclusion

This study identified lower BMI, regular corticosteroid use, xerosis on the legs, and lower Barthel Index as predictors of skin tear development in elderly nursing home residents. These findings provide a practical framework for assessing skin tear risk in long-term care settings. Results indicate that tailored skincare, optimization of nutritional status, and appropriate measures to minimize friction and shear during repositioning and transfers may mitigate the risk of skin tear development in this population. However, further validation through larger, prospective studies is necessary to confirm these results and refine the model for broader clinical application. Future research should also explore additional risk factors that were not captured in this study.

## Electronic supplementary material

Below is the link to the electronic supplementary material.

Supplementary Material 1

Supplementary Material 2

## Data Availability

The data that support the findings of this study are available from the corresponding author upon reasonable request.
