# Supplementary material for: Development of a multivariable prognostic prediction model for skin tears in older nursing home residents
Source: Sci Rep. 2025 Apr 3;15:11373. doi: 10.1038/s41598-025-95944-5 (PMC11965281; doi:10.1038/s41598-025-95944-5)
Supplement: Supplementary file 2 — Supplementary Material 2 [file 41598_2025_95944_MOESM2_ESM.pdf]

Appendix 2: Correlation Matrix

Note: All values represent Pearson correlation coefficients, based on a sample size of n = 101.

	Newly developed Skin Tears at W12?	Age	BMI	Duration_residency	Braden_Scale_W0	Braden_Mobility_W0	Barthel_Index_W0	Barthel_Standing_Mobility_W0	Barthel_Index_Transfer_W0	Sex	BMI_Underweight	BMI_Overweight	Care_level_II_III	Care_level_IV_V	Smoker	Incontinence_Urinary_W0	Incontinence_Faecal_W0	Dementia	Medication_Angiotensin	Medication_Corticosteroids	Polypharmacy	GDS_1_to_3_W0	GDS_4_to_7_W0	Xerosis_Arms_W0	Xerosis_Legs_W0	IAD_W0	PU_W0	Intertrigo_W0	Diabetes	Hemiparesis	Paralytic_Symptoms
Newly developed Skin Tears at W12?	1	-0.033	-0.335	-0.176	-0.227	-0.138	-0.164	-0.113	-0.057	-0.140	0.241	-0.273	-0.064	0.064	0.124	-0.042	0.159	0.160	-0.139	0.032	-0.010	-0.224	0.224	0.112	0.098	-0.070	0.032	-0.001	-0.135	0.140	0.065
Age	-0.033	1	-0.190	-0.129	0.141	0.070	0.062	0.117	0.056	0.255	0.158	-0.054	0.058	-0.058	-0.187	0.031	0.010	0.100	0.158	0.003	-0.127	0.002	-0.002	-0.048	-0.086	0.086	0.088	-0.035	0.111	-0.042	-0.130
BMI	-0.335	-0.190	1	0.127	0.105	0.067	-0.025	0.020	-0.041	0.167	-0.359	0.719	0.117	-0.117	-0.198	0.114	-0.090	-0.125	0.320	0.005	0.047	0.212	-0.212	-0.038	0.018	0.086	-0.026	0.189	0.195	-0.107	-0.078
Duration_residency	-0.176	-0.129	0.127	1	-0.212	-0.227	-0.143	-0.215	-0.188	0.009	-0.139	0.108	-0.176	0.176	-0.078	-0.008	0.032	-0.120	0.000	-0.021	0.209	0.037	-0.037	-0.296	-0.269	-0.070	0.067	0.128	0.035	0.088	0.177
Braden_Scale_W0	-0.227	0.141	0.105	-0.212	1	0.912	0.840	0.848	0.790	0.098	-0.073	0.098	0.388	-0.388	-0.010	-0.362	-0.539	-0.230	0.116	-0.020	0.076	0.388	-0.388	0.107	0.179	-0.021	-0.050	0.038	0.046	-0.404	-0.175
Braden_Mobility_W0	-0.138	0.070	0.067	-0.227	0.912	1	0.811	0.803	0.776	0.032	-0.040	0.103	0.353	-0.353	0.102	-0.350	-0.537	-0.236	0.144	-0.009	0.102	0.334	-0.334	0.087	0.137	0.007	-0.076	0.080	0.037	-0.349	-0.198
Barthel_Index_W0	-0.164	0.062	-0.025	-0.143	0.840	0.811	1	0.842	0.854	0.030	-0.045	-0.017	0.473	-0.473	-0.015	-0.521	-0.697	-0.248	0.076	0.025	0.059	0.468	-0.468	0.113	0.182	-0.089	-0.067	-0.006	-0.015	-0.375	-0.154
Barthel_Standing_Mobility_W0	-0.113	0.117	0.020	-0.215	0.848	0.803	0.842	1	0.765	-0.004	-0.104	0.039	0.370	-0.370	0.029	-0.350	-0.446	-0.088	0.087	-0.039	-0.022	0.298	-0.298	0.148	0.167	0.010	-0.039	0.112	0.020	-0.363	-0.140
Barthel_Index_Transfer_W0	-0.057	0.056	-0.041	-0.188	0.790	0.776	0.854	0.765	1	0.027	0.000	-0.002	0.385	-0.385	0.008	-0.403	-0.478	-0.158	0.066	-0.011	0.101	0.262	-0.262	0.051	0.111	-0.088	-0.113	0.029	-0.018	-0.347	-0.200
Sex	-0.140	0.255	0.167	0.009	0.098	0.032	0.030	-0.004	0.027	1	0.163	0.136	0.116	-0.116	-0.336	0.102	-0.037	0.107	0.050	-0.070	-0.026	0.043	-0.043	-0.091	-0.132	0.071	-0.178	0.035	0.147	-0.179	0.125
BMI_Underweight	0.241	0.158	-0.359	-0.139	-0.073	-0.040	-0.045	-0.104	0.000	0.163	1	-0.276	-0.046	0.046	-0.062	-0.049	-0.007	0.016	0.016	-0.046	0.076	0.035	-0.035	-0.001	-0.057	0.101	-0.046	-0.155	-0.025	0.102	0.229
BMI_Overweight	-0.273	-0.054	0.719	0.108	0.098	0.103	-0.017	0.039	-0.002	0.136	-0.276	1	0.149	-0.149	-0.156	0.164	-0.121	-0.125	0.250	-0.039	-0.004	0.184	-0.184	-0.107	0.070	0.094	0.064	0.216	0.189	-0.056	-0.093
Care_level_II_III	-0.064	0.058	0.117	-0.176	0.388	0.353	0.473	0.370	0.385	0.116	-0.046	0.149	1	-1.000	0.033	-0.192	-0.527	-0.474	0.021	-0.085	0.067	0.617	-0.617	0.100	0.087	-0.221	0.025	-0.193	-0.081	-0.202	-0.010
Care_level_IV_V	0.064	-0.058	-0.117	0.176	-0.388	-0.353	-0.473	-0.370	-0.385	-0.116	0.046	-0.149	-1.000	1	-0.033	0.192	0.527	0.474	-0.021	0.085	-0.067	-0.617	0.617	-0.100	-0.087	0.221	-0.025	0.193	0.081	0.202	0.010
Smoker	0.124	-0.187	-0.198	-0.078	-0.010	0.102	-0.015	0.029	0.008	-0.336	-0.062	-0.156	0.033	-0.033	1	0.054	0.006	-0.250	-0.064	0.175	0.140	0.168	-0.168	0.027	0.152	0.130	0.203	0.011	-0.072	0.163	0.051
Incontinence_Urinary_W0	-0.042	0.031	0.114	-0.008	-0.362	-0.350	-0.521	-0.350	-0.403	0.102	-0.049	0.164	-0.192	0.192	0.054	1	0.324	0.108	0.020	-0.225	-0.028	-0.232	0.232	-0.117	-0.050	0.060	0.078	0.071	-0.005	0.113	0.067
Incontinence_Faecal_W0	0.159	0.010	-0.090	0.032	-0.539	-0.537	-0.697	-0.446	-0.478	-0.037	-0.007	-0.121	-0.527	0.527	0.006	0.324	1	0.359	0.012	0.035	-0.191	-0.671	0.671	-0.186	-0.125	0.041	-0.068	0.073	0.035	0.273	0.089
Dementia	0.160	0.100	-0.125	-0.120	-0.230	-0.236	-0.248	-0.088	-0.158	0.107	0.016	-0.125	-0.474	0.474	-0.250	0.108	0.359	1	0.005	0.056	-0.230	-0.562	0.562	0.068	-0.038	0.097	-0.049	0.056	0.089	-0.071	0.109
Medication_Angiotensin	-0.139	0.158	0.320	0.000	0.116	0.144	0.076	0.087	0.066	0.050	0.016	0.250	0.021	-0.021	-0.064	0.020	0.012	0.005	1	0.076	0.110	0.101	-0.101	0.014	-0.014	0.173	0.178	0.040	0.282	-0.112	-0.082
Medication_Corticosteroids	0.032	0.003	0.005	-0.021	-0.020	-0.009	0.025	-0.039	-0.011	-0.070	-0.046	-0.039	-0.085	0.085	0.175	-0.225	0.035	0.056	0.076	1	0.067	0.008	-0.008	-0.154	0.075	-0.107	-0.041	0.080	0.120	-0.060	0.264
Polypharmacy	-0.010	-0.127	0.047	0.209	0.076	0.102	0.059	-0.022	0.101	-0.026	0.076	-0.004	0.067	-0.067	0.140	-0.028	-0.191	-0.230	0.110	0.067	1	0.249	-0.249	0.085	0.083	0.095	0.067	-0.059	0.170	-0.148	0.058
GDS_1_to_3_W0	-0.224	0.002	0.212	0.037	0.388	0.334	0.468	0.298	0.262	0.043	0.035	0.184	0.617	-0.617	0.168	-0.232	-0.671	-0.562	0.101	0.008	0.249	1	-1.000	0.205	0.124	0.048	0.123	-0.166	-0.077	-0.071	0.106
GDS_4_to_7_W0	0.224	-0.002	-0.212	-0.037	-0.388	-0.334	-0.468	-0.298	-0.262	-0.043	-0.035	-0.184	-0.617	0.617	-0.168	0.232	0.671	0.562	-0.101	-0.008	-0.249	-1.000	1	-0.205	-0.124	-0.048	-0.123	0.166	0.077	0.071	-0.106
Xerosis_Arms_W0	0.112	-0.048	-0.038	-0.296	0.107	0.087	0.113	0.148	0.051	-0.091	-0.001	-0.107	0.100	-0.100	0.027	-0.117	-0.186	0.068	0.014	-0.154	0.085	0.205	-0.205	1	0.355	0.202	0.101	-0.142	-0.044	-0.038	-0.059
Xerosis_Legs_W0	0.098	-0.086	0.018	-0.269	0.179	0.137	0.182	0.167	0.111	-0.132	-0.057	0.070	0.087	-0.087	0.152	-0.050	-0.125	-0.038	-0.014	0.075	0.083	0.124	-0.124	0.355	1	0.194	0.075	-0.013	-0.033	-0.006	-0.116
IAD_W0	-0.070	0.086	0.086	-0.070	-0.021	0.007	-0.089	0.010	-0.088	0.071	0.101	0.094	-0.221	0.221	0.130	0.060	0.041	0.097	0.173	-0.107	0.095	0.048	-0.048	0.202	0.194	1	0.139	-0.050	0.095	0.112	0.049
PU_W0	0.032	0.088	-0.026	0.067	-0.050	-0.076	-0.067	-0.039	-0.113	-0.178	-0.046	0.064	0.025	-0.025	0.203	0.078	-0.068	-0.049	0.178	-0.041	0.067	0.123	-0.123	0.101	0.075	0.139	1	-0.138	0.222	0.128	-0.036
Intertrigo_W0	-0.001	-0.035	0.189	0.128	0.038	0.080	-0.006	0.112	0.029	0.035	-0.155	0.216	-0.193	0.193	0.011	0.071	0.073	0.056	0.040	0.080	-0.059	-0.166	0.166	-0.142	-0.013	-0.050	-0.138	1	0.104	-0.042	0.006
Diabetes	-0.135	0.111	0.195	0.035	0.046	0.037	-0.015	0.020	-0.018	0.147	-0.025	0.189	-0.081	0.081	-0.072	-0.005	0.035	0.089	0.282	0.120	0.170	-0.077	0.077	-0.044	-0.033	0.095	0.222	0.104	1	0.026	0.074
Hemiparesis	0.140	-0.042	-0.107	0.088	-0.404	-0.349	-0.375	-0.363	-0.347	-0.179	0.102	-0.056	-0.202	0.202	0.163	0.113	0.273	-0.071	-0.112	-0.060	-0.148	-0.071	0.071	-0.038	-0.006	0.112	0.128	-0.042	0.026	1	-0.051
Paralytic_Symptoms	0.065	-0.130	-0.078	0.177	-0.175	-0.198	-0.154	-0.140	-0.200	0.125	0.229	-0.093	-0.010	0.010	0.051	0.067	0.089	0.109	-0.082	0.264	0.058	0.106	-0.106	-0.059	-0.116	0.049	-0.036	0.006	0.074	-0.051	1
